# Assessing the efficacy of simulation-based education for paramedics in extended focused assessment with sonography for trauma under physician guidance

**DOI:** 10.1038/s41598-024-54779-2

**Published:** 2024-02-20

**Authors:** Akiko Ohira, Jota Maki, Kohei Ageta, Hikari Nakato, Hikaru Oba, Tomohiro Mitoma, Sakurako Mishima, Kazumasa Tani, Satoe Kirino, Eriko Eto, Atsunori Nakao, Hisashi Masuyama

**Affiliations:** Department of Obstetrics and Gynecology, and Department of Emergency and Disaster Medicine, Okayama University Graduate School of Medicine, Dentistry and Pharmaceutical Sciences, 2-5-1 Shikada-Cho, Kita-Ku, Okayama-Shi, Okayama, Japan

**Keywords:** Simulation-based education, Ultrasound, Paramedics, FAST, Ultrasonography, Health care, Health policy

## Abstract

We investigated the effectiveness of simulation-based education in Focused Assessment with Sonography for Trauma (FAST) to increase the number of Emergency Medical Technicians (EMTs) capable of performing ultrasound examinations in vehicles under the guidance of a physician. Twenty-eight paramedics watched a 14-min video on the features of the ultrasound system, its use, and the scanning method for each part of the body. Each participant performed four FAST examinations using a portable ultrasound device, and the task performance was rated using the Task Specific Checklist (TSC) and Global Rating Scale (GRS). The time required for visualizing each examination site and each FAST was assessed. The mean time required for the first and fourth FAST was 144.6 ± 52.4 s and 90.5 ± 31.0 s, respectively. The time required for each test significantly decreased with repeated testing (p < 0.001). The time to complete FAST was significantly shortened for the pericardial cavity (33.4 ± 23.1/15.3 ± 10.6 s, p < 0.01), right thoracic cavity (25.2 ± 11.8/12.1 ± 8.3 s, p < 0.01), Morrison fossa (19.1 ± 10.8/10.8 ± 6.3 s, p < 0.05), and left thoracic cavity (19.0 ± 8.3/15.6 ± 8.3 s, p < 0.05). TSC and GRS scores were elevated, and all EMTs could obtain valid images. The combination of a brief video lecture and hands-on training significantly reduced the time required for FAST performance. Moreover, repeated practice enabled the EMTs to efficiently obtain accurate and clinically useful images.

## Introduction

With technological advances, ultrasound systems have become more compact and portable, enabling their use at the bedside, particularly in emergency situations. Ultrasonography can detect free abdominal fluid with a sensitivity of 90–95% and a specificity of 90–100%^1,2^.

In recent years, point-of-care ultrasonography (POCUS), a type of ultrasonography that can be performed at the bedside by medical personnel to improve clinical decisions and techniques, has gained global acceptance^3^. Prehospital POCUS was defined in 2011 as one of the top five research priorities in prehospital clinical care provided by physicians. The utility of prehospital POCUS is currently being demonstrated and is considered feasible^4^. A study demonstrated that emergency medical technicians and paramedics performed chest ultrasound examinations on patients in prehospital clinical settings with image quality adequate to determine the presence or absence of lesions^5^.

Focused Assessment with Sonography for Trauma (FAST) is used during initial trauma examinations to identify pericardial effusions, hemothoraces, and intra-abdominal hemorrhage, and its utility has been widely proven in prehospital emergencies^3^. Specifically, its use has been associated with shorter times to surgery, fewer computed tomography studies, shorter hospital stays, fewer complications, and lower costs than those with other assessments^6^. Real-time wireless transmission provides reliable images without compromising image quality or legibility unlike digitally recorded images at the inspection site^7^. However, it is believed that extensive training through a combination of theoretical learning, hands-on training, and clinical experience is needed to develop expertise in POCUS^4^.

In some areas of Japan, transporting a patient to an emergency medical center may require up to an hour, and the situation is even more difficult in severe cases. A demonstration is underway for the regulatory reform to promote legal revision of paramedic practice as a solution to poor medical care in mountainous regions with a declining birthrate and aging population. Kibichuo in the Okayama prefecture was recently designated as a special zone for digital rural health by the Advisory Council for National Strategic Special Zones and other organizations aiming to revitalize rural areas^8^. A regulatory reform demonstration is currently underway in the Digital Rural Special Zone to promote legal revisions of paramedic practice as a solution to poor medical care in mountainous regions with declining birthrates and aging populations.

During emergency transport, paramedics perform examinations and procedures in ambulances and capture patient information using onboard cameras. These data are transmitted remotely and in real time to the doctor at the destination, who verifies the technical effectiveness of the system.

Implementing the aforementioned processes will enable physicians to make diagnostic inferences and prepare for medical treatment and surgical procedures at the receiving institution. Moreover, it will allow EMTs to safely perform specific procedures, which may lead to the prompt provision of medical care upon arrival at the hospital. Ultrasound examinations by EMTs are not currently permitted in Japan; a high-quality educational program is required to implement this service. In addition, only a few reports on ultrasound examinations by EMTs exist globally^9–12.^

In this study, we investigated the effectiveness of simulation education in FAST examinations by paramedics under the guidance of a physician.

## Results

Twenty-eight EMTs participated. The participants comprised 27 men and one woman (mean age, 40.5 ± 5.9 years; mean length of service, 10.1 ± 5.5 years) (Table 1). Four emergency physicians and four obstetricians/gynecologists provided guidance.

**Table 1:** Baseline characteristics of the 28 participants.

Eligibility (n = 28)	
Participants age	40.5 ± 5.9
Years of continuous service of EMT	10.1 ± 5.5
Male	27 (96%)

Data are expressed as number (percentage) or mean ± standard deviation values.

*EMT;* Emergency Medical Technician.

**Figure 1:**
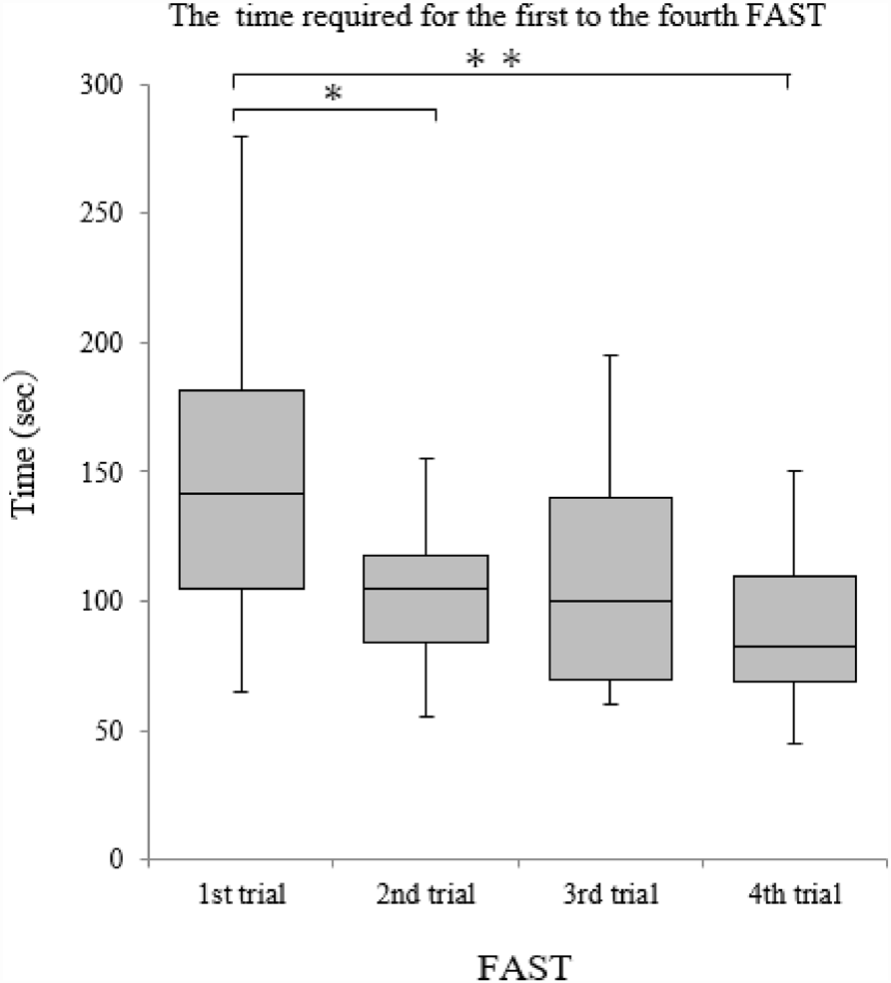
Time required for the first to fourth FAST procedures. FAST, Focused Assessment with Sonography for Trauma (Wilcoxon’s signed rank tests modified by Bonferroni’s method, *p < 0.05, **p < 0.01).

The mean time required for the first to the fourth FAST was 144.6 ± 51.4, 103.9 ± 25.7, 113.6 ± 53.1, and 90.5 ± 31.0 s, respectively. (Fig. 1). Upon repeating the process, we observed significant reductions in time between the first and second tests and between the first and fourth tests. The time required for FAST was significantly shortened by repeating the procedure (*p* = 0.014, 0.003) (Table 2).

**Table 2:** Examination by number of measurements.

Number of measurements		Z score	*p*-value
1st	2nd	− 3.041	0.014
1st	3rd	− 1.993	0.277
1st	4th	− 3.497	0.003
2nd	3rd	− 0.337	4.418
2nd	4th	− 2.268	0.140
3rd	4th	− 1.919	0.330

Wilcoxon’s signed rank tests with Bonferroni’s correction method.

The time required to visualize each of the six FAST test areas was also compared between the first and the fourth examinations (Table 3). Significant time reductions were observed for pericardial cavity (33.4 ± 23.1/15.3 ± 10.6 s; *p* = 0.009), right thoracic cavity (25.2 ± 11.8/12.1 ± 8.3 s; *p* = 0.027), Morrison fossa (19.1 ± 101.8/10.8 ± 6.3 s; *p* = 0.014), and left thoracic cavity (19.0 ± 8.3/15.6 ± 8.3 s;* p* = 0.014) examinations. Multiple regression analysis of reductions in FAST time and site examination time showed that the reduction in time of examination for all sites was significantly related to the reduction in FAST time (Table 4).

**Table 3:** Inspection time by site.

	I	II	III	IV	V	VI
	Pericardial space	Right thoracic cavity	Morrison pouch	Left thoracic cavity	Perisplenic area	Douglas pouch
1st trial	33.4 ± 23.1	25.2 ± 11.8	19.1 ± 10.8	19.0 ± 8.3	21.5 ± 11.8	26.2 ± 14.8
4th trial	15.3 ± 10.6	12.1 ± 8.3	10.8 ± 6.3	15.6 ± 8.3	13.5 ± 9.6	23.1 ± 14.4
*p*	0.009	0.027	0.014	0.036	0.088	2.630

Inspection time (seconds) is expressed as mean ± standard deviation.

Wilcoxon’s signed rank tests with Bonferroni’s correction method.

**Table 4:** Multiple regression analysis of FAST time reduction and site examination time reduction.

Reduction time	B	β	*p*-value	95% CI	
Pericardial space (I)	0.997	0.406	0.000	0.985	1.009
Right thoracic cavity (II)	1.009	0.248	0.000	0.989	1.03
Morrison pouch (III)	0.994	0.23	0.000	0.976	1.013
Left thoracic cavity (IV)	1.014	0.191	0.000	0.992	1.036
Perisplenic area (V)	0.998	0.244	0.000	0.977	1.019
Douglas pouch (VI)	1	0.322	0.000	0.987	1.014

*CI;* confidence interval, *FAST;* focused assessment with sonography for trauma.

**Figure 2:**
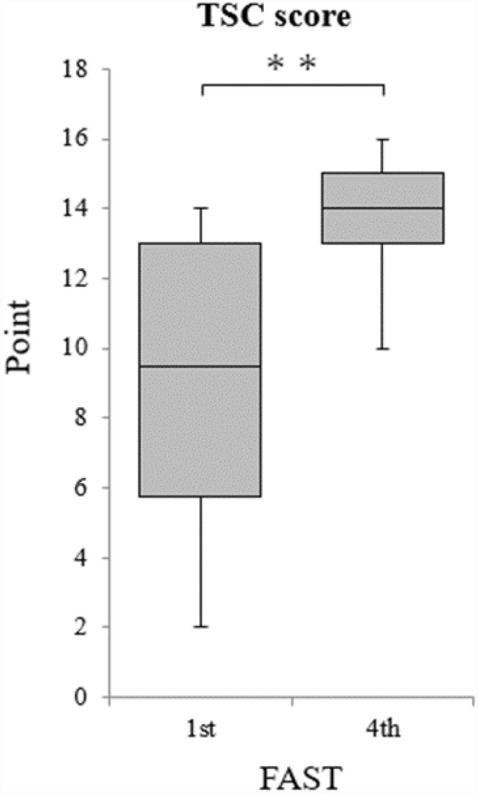
TSC scores for the first to fourth FAST procedures. FAST, Focused Assessment with Sonography for Trauma; TSC, Task-Specific Checklist (T-tests, **p < 0.01).

**Figure 3:**
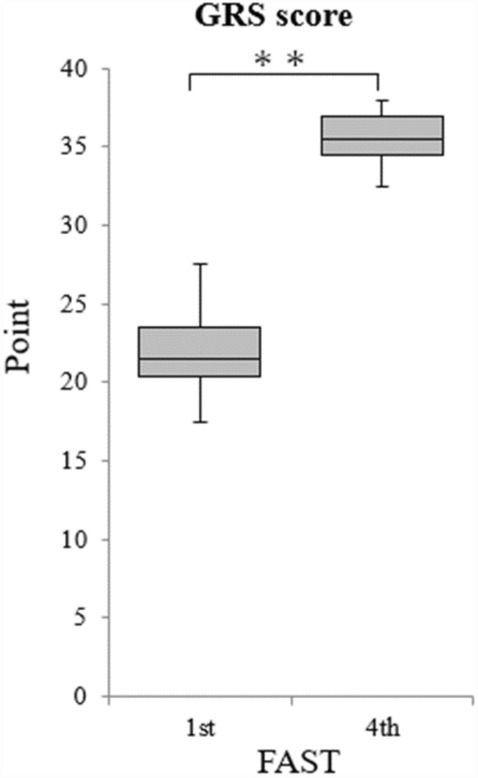
GRS scores for the first to fourth FAST procedures. FAST, Focused Assessment with Sonography for Trauma; GRS, Global Rating Scale (T-tests, **p < 0.01).

The achievement and quality aspects of the FAST procedures performed by the 28 paramedics were evaluated using the TSC and GRS, respectively. TSC and GRS scores were evaluated for the first and fourth sessions (Figs. 2 and 3). TSC scores for the first and fourth sessions were 9.2 ± 4.0 and 13.5 ± 2.1, respectively, indicating that the fourth session was significantly more successful than the first session (*p* = 0.000). GRS scores for the first and fourth ultrasound sessions were 21.8 ± 2.2 and 35.5 ± 1.8, respectively, indicating that the fourth ultrasound examination was also superior in quality (p = 0.000). The k coefficients were as follows: κ = 0.00 (2nd order weights) and κ = 0.22 (2nd order weights) for the first and fourth sessions, respectively.

## Discussion

In this study, the total time required for FAST was approximately 1.5 min, which was approximately 30 s shorter than that described in existing reports^10^. By repeating the process several times, the time required for FAST was reduced to approximately 90 s.

As for the instruction time, as only FAST was performed as the ultrasound examination, it was considered that the training time should be short. Reportedly, training provided to EMTs on echocardiography and pulmonary echocardiography could be completed in 1–4 h^9–11,13^. Monti et al. also reported an overall improvement in the extended-FAST knowledge of 34 medics after approximately 4 h of combined lectures and hands-on training^14^. In this study, a similar combination of videos and practical training was used. In addition, the training can be conducted with the cooperation of other personnel while on duty. Based on our assessment, clinical training at a medical institution may not be necessary. In this study, approximately two hours of training was considered acceptable.

The examination can be performed during transportation and does not lead to an extension of the transportation time. However, it is suggested that measurement should be performed while the vehicle is stopped, which may increase the time by approximately 2 min^12^. Nonetheless, reports from other countries do not typically consider transportation times of 1 h as a standard^15^ and in mountainous areas such as ours, further investigations are necessary.

The usefulness of FAST in prehospital emergency care has been widely demonstrated. Reportedly, prehospital emergency ultrasound findings led to a change in treatment in 49 of 99 patients (49.5%; 38.7% in the trauma group and 54.4% in the non-trauma group)^16^ In other studies, ultrasound examination resulted in changes in transport destination, transport priority, and monitoring requirements (e.g., no need for physician accompaniment) in 33 of 99 patients (16/31 trauma patients and 17/68 non-trauma patients)^6,17,18^. Regarding the examination sites, reductions in the imaging times for the pericardial cavity, right thoracic cavity, Morrison pouch, and left thoracic cavity had influenced the shortening of FAST completion times. However, it is unclear whether the shorter examination times for the pericardial cavity are attributed to the improved proficiency in applying the difficult ultrasound technique (owing to repeated use) or to the time taken to familiarize with the technique since it was the first area to be examined. Similarly, whether the reason for the short examination times for the right thoracic cavity, Morrison pouch, and left thoracic cavity was that these right thoracic cavity areas were also examined during the first half of the examination period as in the case of the pericardial cavity, thus getting sufficient time to be familiarized with the technique, is not clear. In the future, it is necessary to establish an educational program, implement the technique in moving ambulances, develop a simple transmission system, and address economic aspects.

The study had some limitations. This was a single-center study. The results cannot be generalized, and care must be taken to ensure that EMTs across the country acquire training on uniform FAST techniques. Moreover, repeated instructions may be necessary to achieve a certain level of skills. There might have been differences in the method and duration of FAST among the four groups based on their years of experience, their teaching by emergency physicians/obstetricians, and their teaching/evaluation methods. Furthermore, as ultrasound devices by various manufacturers were used, discrepancies in the examination procedures might have occurred depending on the equipment used. Moreover, the ultrasonography simulator used in this study only provides normal findings, so further studies are required to learn to detect abnormal findings. This was only a simulation study, and further study is needed to determine the effects of the actual ambulance environment, states of the examination sites, constrained conditions, and internet connection conditions.

It is also necessary to consider the practices of EMTs not only in our country but also in other countries around the world.

Paramedics are generally qualified to perform only limited measures in the prehospital setting. By performing only limited procedures, their own liability is more protected and their legal safety is assured. The procedures that can be performed vary from country to country. In Japan, since 2022, previously prehospital procedures can also be performed in emergency rooms, thus shifting the tasks of different medical personnel.

For paramedics, it is often highly challenging to perform diagnostic imaging. Therefore, it is necessary to construct an Internet of Things that can transmit the status of paramedics to the receiving physician in real time, and enable appropriate treatment. There is valuable evidence for the autonomous assessment and management of patients by paramedics, which is necessary in order to reduce Emergency Department conveyance^19^.

The establishment of an educational system for other EMTs (i.e. via simulations) and the design of a system that would expand the scope of what paramedics are permitted to do (and thereby, improve their social standing based on metrics such as salary), would require a task shift for various medical professionals. Globally, regulatory obstacles still impede the broader application of EMT demonstrations.^20^ Future initiatives should include FAST demonstrations for EMTs utilizing the simulation techniques described in our study, with potential for international expansion. Furthermore, this report may play a crucial role in supporting the case for expanding EMT services worldwide. Evidence from this study could facilitate further demonstrations, such as training courses for EMTs on ultrasound techniques with healthy subjects or research on the use of ultrasound in real emergency scenarios.

In addition, the results were not reliable because two contrasting evaluations of the GRS were observed, but this may be due to the fact that one emergency medicine specialist and one obstetrics and gynecology specialist (and a Japanese ultrasound specialist) were the scorers in this case, which may have caused differences in opinion in the scorers' subjective evaluation of the scores.

This study showed that short video lectures and practical training using an ultrasound simulator under physician guidance significantly reduced the time required by EMTs to perform FAST. In particular, the time required to visualize the pericardial and right thoracic cavities was considered to influence the overall reduction in the time required to perform FAST. Moreover, accurate images that could be utilized by physicians were obtained through repeated procedures. The time required for the FAST examination was comparable to that reported previously, suggesting that information could be collected in a short time during long transport periods. Based on the results of this study, we aim to continue investigating these issues and work towards rendering ultrasound examinations by paramedics under remote physician guidance possible during transport.

## Methods

### Study population

All procedures were conducted in accordance with the ethical standards of the Declaration of Helsinki. This prospective study was approved by the Research Ethics Committee of Okayama University School of Medicine (protocol #2306-015), which waived the need for obtaining informed consent from each participant. This research falls under the category of "research that does not use samples obtained from the human body" under the Ethical Guidelines. However, if informed consent is not obtained, the researcher will notify the research participants or disclose information about the research, including the purpose of use of the information used in the research, and will ensure that the research participants have the opportunity to refuse the implementation or continuation of the research. The research participants are guaranteed the opportunity to reject the research. For this purpose, the documents approved by the Committee for this research are posted on the hospital's website.

Paramedics who volunteered for the simulation education participated in the training. The paramedics did not have any prior training in FAST. We invited EMTs from municipalities undergoing regulatory reform, designated by the Cabinet Office, who were available to participate in the training day. Japanese paramedics with up to 25 years of work experience, no previous ultrasound experience, and no physical limitations in performing ultrasound examinations were included in the study.

Practical guidance for FAST was provided by an emergency physician or obstetrician/gynecologist with expertise in ultrasound examinations. All physicians had at least 7 years of post-graduation experience, were board certified, and had performed hundreds of abdominal ultrasound examinations per year. Emergency physicians routinely provide trauma care and obstetricians routinely respond to cases of hemorrhagic shock, such as in ectopic pregnancies; therefore, they are familiar with employing the FAST test on a daily basis.

### Intervention

The training should cover several aspects, such as the identification of patients who should undergo rapid and simplified ultrasonography, probe application, examination of patients based on their medical conditions, and basic interpretation of images. Moreover, direct instruction by a medical professional and a hands-on training course on the actual probe application are recommended^12^. In the present study, the physician provided direct instructions on how to apply the probe and interpret the images.

A 2-h hands-on training session was held at our hospital in December 2022.

**Figure 4:**
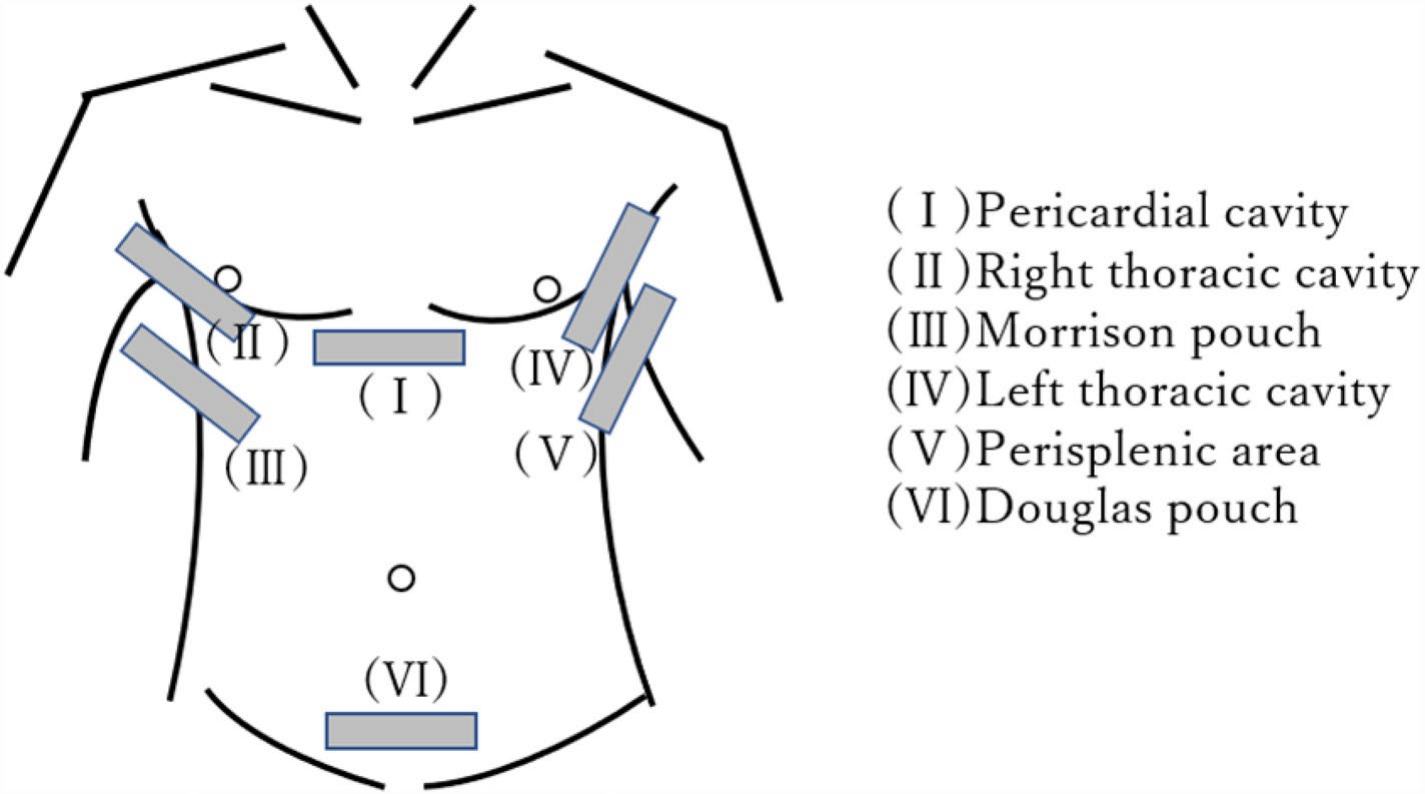
Focused Assessment with Sonography for Trauma.

**Figure 5:**
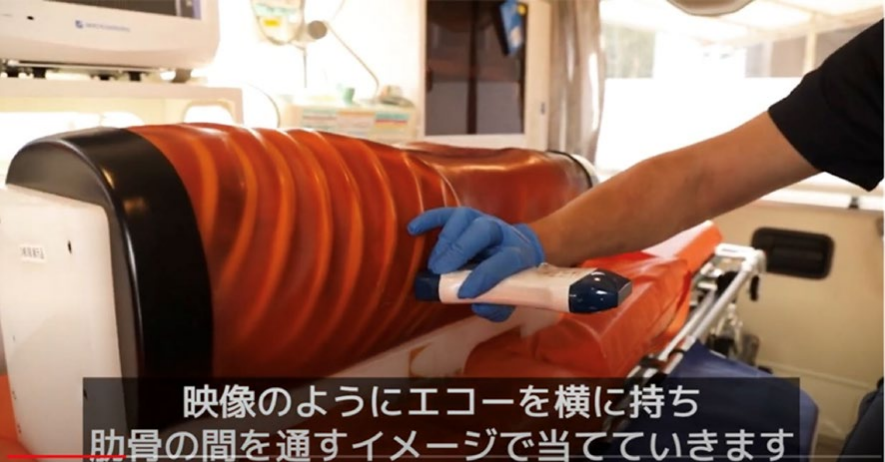
Educational video (in Japanese).

At the beginning of the session, a 14-min video regarding the principles, features, and usage of ultrasonography equipment, as well as demonstrating how to operate the equipment and interpret ultrasound images, was presented. In addition, the participants were briefed regarding FAST, the six anatomical areas to be assessed during FAST, and how these areas appear on ultrasound images (Figs. 4 and 5).

**Figure 6:**
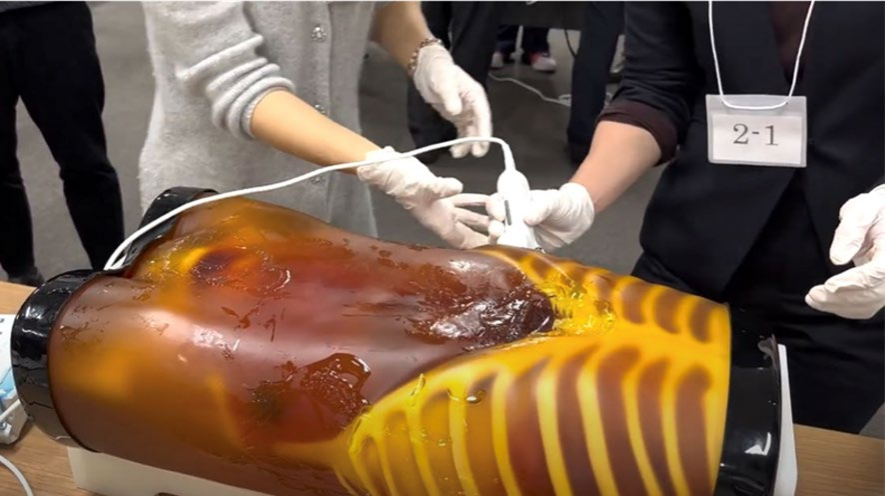
Ultrasound guidance.

**Figure 7:**
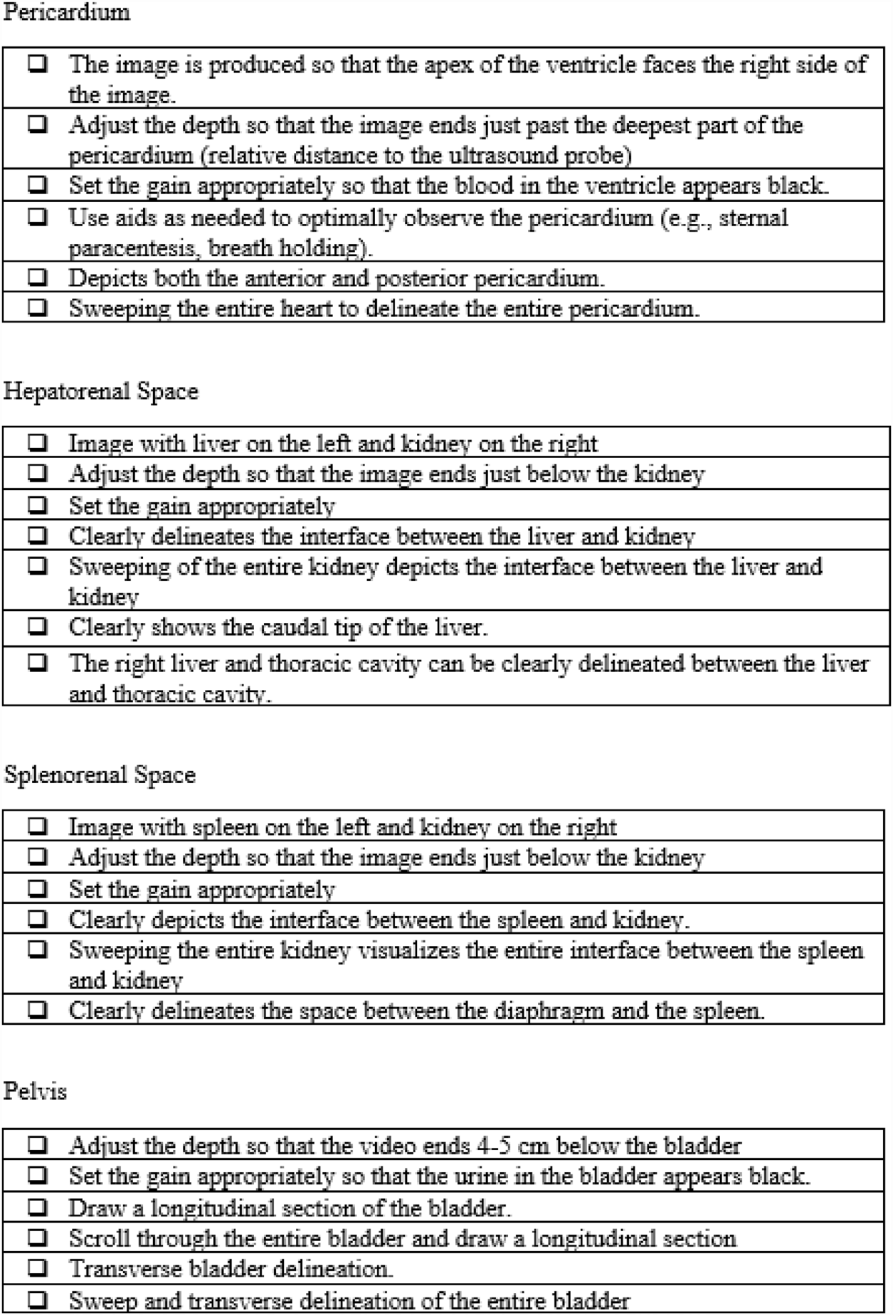
Task-Specific Checklist.

Subsequently, the participants were divided into four groups of seven. Under the guidance of a physician, the EMTs underwent practical training in FAST using portable ultrasonography equipment to identify the presence of fluid accumulation in the (I) pericardial cavity, (II) right thoracic cavity, (III) Morrison pouch, (IV) left thoracic cavity, (V) perisplenic region, (VI) Douglas pouch, and other sites (Fig. 6). Four FAST procedures were performed by each participant, and the time required for each procedure at each examination site was measured. During the FAST procedures, each procedure was followed by a technique review by the supervising physician. The proficiency of FAST was evaluated by two physicians (AO and KA) using a Task-Specific Checklist (TSC) (Fig. 7). At the same time, two people evaluated the video that had been taken. The quality assurance of FAST was evaluated by two physicians (AO and KA) using the Global Rating Scale (GRS) (Table 5)^21^. The TSC provides a binary measure of performing various components of a complicated task (scores: ‘‘1’’ for task accomplishment; ‘‘0’’ for task failure). In the present study, “the space between the right liver and the thoracic cavity can be clearly delineated” was added to the list of 25 items in total—this item was deemed necessary to evaluate right pleural effusion. By contrast, the GRS measures the quality of task performance on a five-point scale.

**Table 5:** Global rating scale.

1	2	3	4	5
Skin contact				
Insufficient amount of gel used, inadequate skin contact of probe		Appropriate amounts of gel are used, and in most cases, adequate skin contact is achieved		Always use the appropriate amount of gel to achieve adequate skin contact
Image Adjustment				
Improper gain or depth settings		Gain and depth are properly adjusted but may need to be adjusted many times during the procedure		Adjust gain and depth to appropriate levels only once at the beginning of each section
Initial probe placement				
Frequent readjustment of the probe position on the skin or only an inadequate field of view is obtained		Can correctly position probe for proper field of view, but needs occasional readjustment		With minimal readjustment, the probe can be correctly positioned to obtain the proper view on the first try
Image sweep				
After the measurement position is fixed, keep changing the position		Once the probe position is established, the movement is almost smooth		After the probe position is established, the probe moves subtly in a smooth sweep
Positioning and probe handling				
Repeatedly assume awkward positions or hold probes in an inconvenient or inappropriate manner		Occasionally, they may assume awkward positions or hold the probe in an inappropriate manner		Assume a comfortable position and hold the probe in an appropriate manner
Execution time (less than 2 min should be considered average)				
Excessive time required to complete the test		Finish the exam in an average amount of time		Finish the exam fast enough to get a passing score
Flow of the procedure (is it done according to the procedure)				
There are many jumps between anatomical areas that are consistently unorganized		Mostly organized, but occasionally jumps between anatomical areas		Move smoothly from area to area to complete the procedure
System of government				
Cannot complete the exam without significant guidance		Able to work accurately with moderate guidance		Able to work independently without direction
Overall performance				
Unacceptable performance, multiple critical deficiencies	Unacceptable performance, some serious defects	Unacceptable performance, minor deficiencies only	Acceptable performance	Outstanding performance, expert FAST performers

The ultrasound equipment used in this study was a Vscan Air™ CL (GE HealthCare Japan Co., Ltd., Tokyo, Japan) with a 2–5-MHz convex probe (SonoAlpha handheld echo c5lc; Taisho Biomed Instruments Co., Ltd., Osaka, Japan), a 3.5–5-MHz convex probe (Lumify; Philips Japan Co., Ltd., Tokyo, Japan), and a 2–5-MHz convex probe (iViz air Ver.5; FUJIFILM Medical Co., Ltd., Tokyo, Japan). The ultrasonography simulator used was FAST/ER FAN (KYOYO KAGAKU Co., Ltd., Kyoto, Japan).

### Outcomes

The primary outcome was the time required for FAST. Four FAST procedures were performed, and the mean measurement time was calculated. The measurement started when the participant placed the ultrasound probe on the simulator until the probe was released from the simulator after the ultrasound of the Douglas fossa was performed. Secondary outcomes were the measurement time for each of the six examination sites, the TSC score, and the GRS score.

### Statistical analyses

Means are listed as the mean ± standard error. T-tests, one-way analysis of variance, Wilcoxon’s signed rank tests modified by Bonferroni’s method, and multiple regression analysis were performed. A p-value < 0.05 was considered statistically significant. The time required for the first to the fourth FAST and whether there were any differences in the time required after repeating the test were based on Wilcoxon's signed rank tests with Bonferroni 's correction method. T-tests were used to evaluate the trends in TSC and GRS. Multiple regression analysis was used for the reductions in the FAST time and site inspection time. For the GRS, each rater was scored individually, and inter-rater reliability was assessed using the kappa coefficient. The analysis was performed using Excel statistics (BellCurve for Excel statistical software program; Social Survey Research Information Co., Ltd., Tokyo, Japan) and SPSS version 26.0 (IBM Co., Ltd., Tokyo, Japan).

### Ethics approval and consent to participate

This prospective study was approved by the Research Ethics Committee of Okayama University Medical Department (protocol number: 2306–015), which waived the requirement for obtaining informed consent from each participant.

## Data Availability

The datasets used and/or analyzed during the current study are available from the corresponding author on reasonable request.
